# Response to: Comment on “Prevalence, Etiology, and Risk Factors of Tinea Pedis and Tinea Unguium in Tunisia”

**DOI:** 10.1155/2018/2563207

**Published:** 2018-05-22

**Authors:** Nourchène Toukabri, Cyrine Dhieb, Dalenda El Euch, Mustapha Rouissi, Mourad Mokni, Najla Sadfi-Zouaoui

**Affiliations:** ^1^Laboratoire de Mycologie, Pathologies et Biomarqueurs, Faculté des Sciences de Tunis, Université Tunis El Manar, 2092 Tunis, Tunisia; ^2^Service de Dermatologie et de Vénéréologie, Hôpital La Rabta, Tunis, Tunisia; ^3^Institut National de la Recherche Agronomique de Tunis, Tunis, Tunisia

We are grateful to Dr. Talel Badri [1] for his comments and suggestions on the article “Prevalence, Etiology, and Risk Factors of Tinea Pedis and Tinea Unguium in Tunisia” [2].

We have carefully looked at all the comments and earnestly hope that the provided responses will fulfill the concerns.

The statistical analysis of predisposing factors of foot mycosis using odds ratio and multivariate analysis “PCA-MCA” is explained in the following paragraph in accordance with Table 1.

Statistical analysis was performed with SPSS software (Statistical Package for Social Sciences version 20.0, SPSS Inc., Armonk, NY). Odds ratio (OR) with 95% confidence interval (CI) was measured. The chi-square (*χ*^2^) was also used to calculate significant differences in characteristics between patients. Differences with *p* < 0.05 were considered statistically significant. Multivariate analyses were carried out by two methods: principal components analysis (PCA) and multivariate correspondence analysis (MCA).

Table 1 shows results of odds ratio, 95% CI, and *P* value; as a conclusion from Table 1, factors associated with foot mycosis such as sex, nail trauma, peripheral vascular disease, psoriasis, and age group over 51 do not present risk factors, whereas presence of dermatological pathology, obesity, wearing used shoes, occlusive shoes, smoking, attending swimming pools, making pedicure, and presence of fungal infection of the skin represent a risk factor of foot mycosis. However, practicing ritual washing, physical activities, attending communal shower, presence of family history, trauma of the nail, application of henna, walking barefoot, using thermal station, presence of associated fingernails, diabetes, and immunosuppressive therapy are protective factors.

Consequently, subjects especially between the age group 40 and 50 seem to be the most exposed to foot mycosis.

Multivariate analysis was carried out to determine the relationship between factors associated with foot mycosis. The correlation between factors, as shown in Table 2, discriminates factors highly related from those having a weak relationship with the infection.

For example, obesity is highly correlated to diabetes and peripheral vascular disease; however, wearing occlusive shoes is not correlated to ritual washing and attending communal shower.

As a result from this correlation matrix and the PCA (principal components analysis), factors are distributed as shown in Figure 1. From this illustration, we conclude that we have three groups of factors: most factors positively close to PC2 axis seem to be risk factors for foot mycosis, whereas most factors positively close to PC1 axis (sex, family history, wearing used shoes, nail trauma, immunosuppressive drugs, season, and peripheral vascular disease) seem to be with no risk; however, the group of factors negatively close to PC1 axis (physical activities, pedicure, occlusive shoes, diabetes, walking barefoot, swimming pools, smoking, thermal station, associated fingernail onychomycosis, dermatological pathology, obesity, fungal infection of the skin, psoriasis, application of henna, and autoimmune disease) are protectors from risk.

We have also carried out a second multivariate analysis which is MCA (multiple correspondence analysis) in order to compare results of the two multivariate methods.

A plot illustrating eignvectors of factors associated with foot mycosis allows to discriminate between two essential groups: the first group gathering the sex, peripheral vascular disease, diabetes, and ritual washing, and the second including psoriasis, autoimmune disease, dermatological pathology, and obesity (Figure 2).

From this work and depending on odds ratio analysis and the two multivariate methods used, we can conclude that we generally obtain the same groups of factors with some difference depending on the performance of methods.

It is true that there is a selection variation in our series. The clinical consultants are much more prone to suggest the mycological test in tinea unguium than tinea pedis because of the duration and the cost of treatment.

In order to respond to the last comment, we have a certitude explanation of nonsignificance of the two factors (ritual washing and communal shower), but it may be related to the improvement of hygiene in these facilities (mosques and hammams) and the individual behavior.

## Figures and Tables

**Figure 1:**
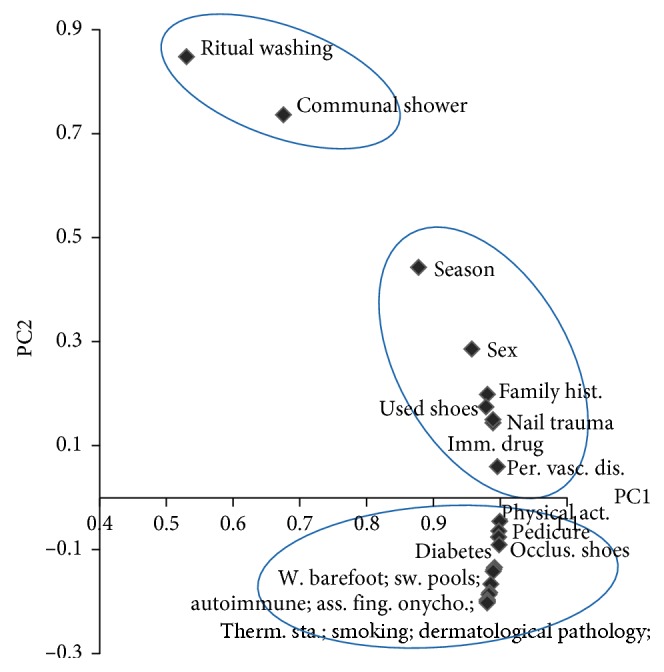
Principal components diagram of factors associated with foot mycosis.

**Figure 2:**
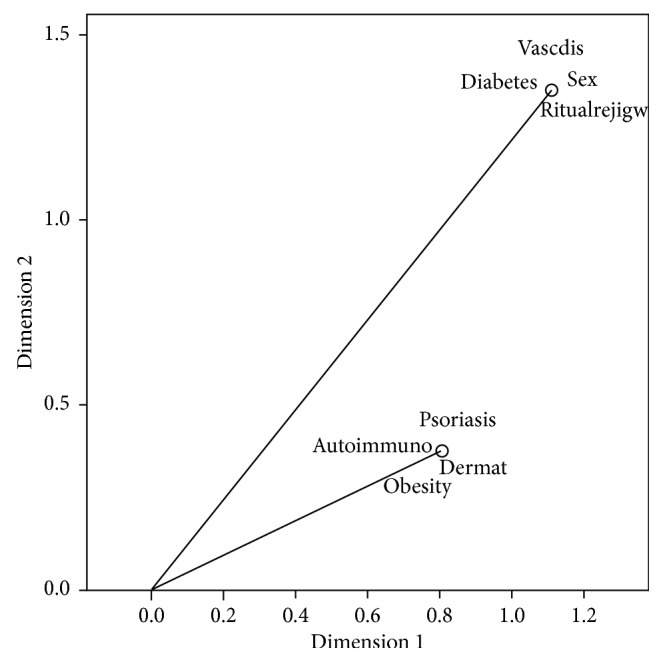
Multiple correspondence analysis of factors associated with foot mycosis.

**Table 1:** Odds ratio of predisposing factors affecting foot mycosis epidemiology.

Predisposing factors	Odds ratio	95% CI	*P* value	Risk type
Sex	1.5635	0.7659 to 3.1916	0.2196	Absent
Ritual washing	0.7660	0.4058 to 1.4459	0.4108	Protector
Diabetes	0.8962	0.3579 to 2.2441	0.8149	Protector
Peripheral vascular disease	1.5683	0.6744 to 3.6467	0.2960	Absent
Autoimmune disease	5.1405	0.3045 to 86.7710	0.2562	Increase
Dermatological pathology	3.1878	0.1848 to 55.0016	0.4250	Increase
Obesity	2.3353	0.1326 to 41.1320	0.5623	Increase
Physical activities	0.4898	0.2379 to 1.0085	0.0527	Protector
Family history	0.6837	0.3596 to 1.2999	0.2461	Protector
Application of henna	0.7941	0.0935 to 6.7479	0.8328	Protector
Communal shower	0.8596	0.4637 to 1.5937	0.6311	Protector
Nail trauma	1.2677	0.6035 to 2.6628	0.5310	Absent
Wearing used shoes	15.702	2.1324 to 115.6224	0.0069	Increase
Season^(a)^	0.0425	0.0129 to 0.1398	0.0001	Protector
Psoriasis	1.7753	0.0984 to 32.0326	0.6973	Absent
Fungal infection of the skin	2.0545	0.1154 to 36.5672	0.6240	Increase
Occlusive shoes	14.391	0.8718 to 237.5528	0.0623	Increase
Swimming pools	8.3218	0.4995 to 138.6327	0.1398	Increase
Smoking	3.7646	0.2201 to 64.3923	0.3601	Increase
Walking barefoot	0.7220	0.2641 to 1.9737	0.5255	Protector
Pedicure	15.474	0.9382 to 255.2066	0.0554	Increase
Thermal station	0.4353	0.1767 to 1.0724	0.0706	Protector
Associated fingernail onychomycosis	0.5434	0.2109 to 1.4003	0.2066	Protector
Immunosuppressive drugs	0.8067	0.4118 to 1.5804	0.5313	Protector

*Age (years)*				
1 to 10	0.1254	0.0245 to 0.6408	0.0126	Protector
11 to 20	0.3458	0.1184 to 1.0095	0.0521	Protector
21 to 30	0.6783	0.3082 to 1.4925	0.3346	Protector
31 to 40	0.9510	0.4373 to 2.0679	0.8991	Protector
41 to 50	2.4662	0.9429 to 6.4505	0.0658	Increase
51 to 60	1.1572	0.5349 to 2.5034	0.7107	Absent
61 to 70	1.0117	0.3768 to 2.7165	0.9816	Absent
>70	2.9077	0.3818 to 22.1439	0.3028	Absent

^(a)^Significance of “spring compared to other seasons (summer  +  autumn  +  winter).”

**Table 2:** Correlation between factors associated with foot mycosis.

Ritual washing	0.751																					
Diabetes	0.927	0.452																				
Peripheral vascular disease	0.973^∗^	0.58	0.988^∗^																			
Dermatological pathology	0.887	0.363	0.995^∗∗^	0.969^∗^																		
Obesity	0.883	0.355	0.994^∗^	0.967^∗^	1.000^∗∗^																	
Physical activities	0.94	0.489	0.999^∗∗^	0.993^∗∗^	0.990^∗^	0.989^∗^																
Family history of foot mycosis	0.994^∗∗^	0.687	0.959^∗^	0.989^∗^	0.926	0.923	0.970^∗^															
Application of henna	0.880	0.351	0.994^∗∗^	0.966^∗^	1^∗∗^	1.000^∗∗^	0.988^∗^	0.921														
Communal shower	0.858	0.983^∗^	0.607	0.718	0.526	0.519	0.639	0.807	0.515													
Nail trauma	0.990^∗^	0.652	0.971^∗^	0.996^∗∗^	0.943	0.940	0.979^∗^	0.998^∗∗^	0.938	0.779												
Wearing used shoes	0.991^∗∗^	0.668	0.958^∗^	0.989^∗^	0.928	0.925	0.964^∗^	0.991^∗∗^	0.922	0.792	0.995^∗∗^											
Season	0.955^∗^	0.835	0.835	0.896	0.779	0.775	0.861	0.949	0.773	0.912	0.928	0.911										
Psoriasis	0.88	0.35	0.994^∗∗^	0.966^∗^	1^∗∗^	1^∗∗^	0.988^∗^	0.921	1^∗∗^	0.514	0.938	0.923	0.772									
Fungal infection of the skin	0.881	0.353	0.994^∗∗^	0.966^∗^	1^∗∗^	1^∗∗^	0.988^∗^	0.922	1^∗∗^	0.517	0.939	0.924	0.773	1^∗∗^								
Occlusive shoes	0.933	0.464	0.999^∗∗^	0.991^∗∗^	0.993^∗∗^	0.992^∗∗^	0.997^∗∗^	0.961^∗^	0.991^∗∗^	0.618	0.974^∗^	0.965^∗^	0.833	0.991^∗∗^	0.992^∗∗^							
Swimming pools	0.909	0.409	0.999^∗∗^	0.981^∗^	0.999^∗∗^	0.998^∗∗^	0.995^∗∗^	0.943	0.998^∗∗^	0.568	0.959^∗^	0.946	0.805	0.998^∗∗^	0.998^∗∗^	0.998^∗∗^						
Smoking	0.889	0.368	0.996^∗∗^	0.971^∗^	1^∗∗^	1^∗∗^	0.990^∗∗^	0.928	1^∗∗^	0.531	0.945	0.93	0.782	1.000^∗∗^	1.000^∗∗^	0.994^∗∗^	0.999^∗∗^					
Walking barefoot	0.909	0.412	0.999^∗∗^	0.981^∗^	0.999^∗∗^	0.998^∗∗^	0.996^∗∗^	0.945	0.998^∗∗^	0.57	0.959^∗^	0.944	0.812	0.998^∗∗^	0.998^∗∗^	0.997^∗∗^	1^∗∗^	0.999^∗∗^				
Pedicure	0.938	0.474	0.999^∗∗^	0.992^∗∗^	0.992^∗∗^	0.991^∗∗^	0.997^∗∗^	0.964^∗^	0.990^∗^	0.627	0.977^∗^	0.968^∗^	0.838	0.990^∗^	0.990^∗∗^	1^∗∗^	0.997^∗∗^	0.992^∗∗^	0.996^∗∗^			
Thermal station	0.905	0.404	0.998^∗∗^	0.978^∗^	0.999^∗∗^	0.998^∗∗^	0.995^∗∗^	0.942	0.998^∗∗^	0.563	0.956^∗^	0.94	0.81	0.998^∗∗^	0.998^∗∗^	0.996^∗∗^	0.999^∗∗^	0.999^∗∗^	1^∗∗^	0.995^∗∗^		
Associated fingernails	0.906	0.406	0.999^∗∗^	0.979^∗^	0.999^∗∗^	0.998^∗∗^	0.996^∗∗^	0.943	0.998^∗∗^	0.565	0.957^∗^	0.942	0.81	0.998^∗∗^	0.998^∗∗^	0.997^∗∗^	0.999^∗∗^	0.999^∗∗^	1^∗∗^	0.996^∗∗^	1^∗∗^	
Immunosuppressive drugs	0.988^∗^	0.646	0.973^∗^	0.996^∗∗^	0.946	0.943	0.982^∗^	0.998^∗∗^	0.942	0.773	1^∗∗^	0.992^∗∗^	0.931	0.941	0.942	0.975^∗^	0.961^∗^	0.947	0.962^∗^	0.978^∗∗^	0.959^∗^	0.960^∗^
	Sex	Ritual washing	Diabetes	PVD	DP	Obesity	PA	FH	Application of henna	CS	Nail trauma	Wearing US	Season	psoriasis	Fungal IS	Occlusive Shoes	Swimming pools	Smoking	WB	Pedicure	Thermal station	AFO

^∗^Correlation is significant at 0.05; ^∗∗^correlation is significant at 0.01; AFO: associated fingernail onychomycosis; CS: communal shower; DP: dermatological pathology; FH: family history; PA: physical activities; PVD: peripheral vascular disease; WB: walking barefoot.
